# Retrospective Analysis of Specimen Quality in Temporal Artery Biopsies for Giant Cell Arteritis and Disease Association in North Midlands, England

**DOI:** 10.7759/cureus.68259

**Published:** 2024-08-30

**Authors:** Adeel Abbas Dhahri, Kamran Hamid, Tomasz A Galus, Chris J Swift, Shazab Islam, Mehvish Adeel Dhahri, Anthony Jaipersad, Sriram Rajagopalan

**Affiliations:** 1 Vascular Surgery, University Hospital North Midlands NHS Foundation Trust, Stoke-on-Trent, GBR; 2 Hospital Medicine, University Hospital North Midlands NHS Foundation Trust, Stoke-on-Trent, GBR; 3 Surgery, Wirral University Hospital, Liverpool, GBR; 4 Psychiatry, University Hospital North Midlands NHS Foundation Trust, Stoke-on-Trent, GBR; 5 Medicine, Walsall Manor Hospital, Walsall, GBR

**Keywords:** clinical audit, gca, tab, giant cell arteritis (gca), temporal artery biopsy

## Abstract

Background

Temporal artery biopsy (TAB) is the recommended index diagnostic method for giant cell arteritis (GCA). Per the British Society for Rheumatology (BSR) guidelines, we assessed our procedural performance. Additionally, we evaluated the occurrence of GCA diagnosis in immunosuppressed patients and other comorbidities.

Methods

Following the audit registration, a retrospective analysis of prospectively collected data was conducted from 2017 to 2022 at a large university hospital in North Midlands, England. Data on demographics and comorbidities were gathered. The study's primary outcome was adherence to BSR guidelines and our service provisions. Secondary outcomes included examining the relationship between biopsy-confirmed GCA and other comorbidities. Statistical analysis was carried out using SPSS version 29 (IBM Corporation, Armonk, New York, United States of America). Two-sample t-test and Chi-square/Fisher exact test were used for continuous and categorical variables, respectively. Holm-Bonferroni method was incorporated to adjust for multiple comparisons.

Results

A total of 156 patients who underwent temporal artery biopsy (TAB) were included in the study, with a male-to-female ratio of 0.44:1. The median age was 73. Among the patients, 19% were smokers. The procedures were performed by either a vascular surgeon (119, 76%) or by an ophthalmologist (37, 24%). Two-thirds of the patients underwent TAB within seven days of referral. In 73, 47% of cases, the post-fixation biopsy sample size exceeded 10 mm. Positive biopsy results were found in 45 patients (29%). GCA was confirmed in 39% of patients with polymyalgia rheumatica (PMR), 24% with diabetics, 20% with hypothyroidism, 29% with hypertension, 32% with hyperlipidaemia, and 26% with other inflammatory diseases. However, the p-value was below the statistically significant threshold. The biopsy outcome was also not dependent on the speciality, time from referral to biopsy, nor on the length of the post-fixation specimen.

Conclusions

Temporal artery biopsy remains a valuable and crucial diagnostic tool in challenging equivocal cases of giant cell arteritis (GCA), although it is limited by its sensitivity, but there is also room for improvement. There is still uncertainty regarding the relationship between biopsy positivity, post-fixation sample size, and the interval between referral and procedure. Additionally, the speciality of the clinician performing the biopsy does not appear to significantly influence the likelihood of a positive result. We still do not fully understand why this is, but the association of the GCA with other comorbidities was unpredictably insignificant.

## Introduction

Giant cell arteritis (GCA) is the most prevalent primary vasculitis affecting large and medium-sized blood vessels, often leading to severe complications such as blindness or stroke. It primarily affects individuals aged 50 and older and is more common in females. The most commonly reported incidence of GCA is in Northern Europe, affecting the white population. In the United Kingdom, the overall age-adjusted incidence rate is 2.2 per 10,000 person-years, with notable regional differences, as the condition is more prevalent in the southern regions than in the northern ones [1-3].

The pathophysiology of GCA involves inflammation within the walls of arteries, which is accompanied by intimal hyperplasia. This ultimately leads to ischaemia in the affected end organs [4]. The diagnosis of GCA is primarily based on clinical criteria, as the symptoms are often non-specific and can include headache, scalp tenderness, jaw claudication, visual loss, and stroke. In one study, 17% of patients with GCA experienced irreversible visual loss, highlighting the need for prompt treatment if the disease is strongly suspected [3-5].

A history of comorbidities and medications that could increase the risk of GCA should be carefully evaluated. Such conditions include autoimmune disorders like polymyalgia rheumatic (PMR), infection, hypertension, hyperlipidaemia, diabetes, hypothyroidism, osteoporosis, fractures, peptic ulcers, and psychiatric effects of medications [4,6].

According to the literature and guidelines, temporal artery biopsy (TAB) is regarded as the gold standard for diagnosing GCA. It is commonly performed in suspected cases of sight-threatening arteritis, especially when ultrasound results are inconclusive. The sensitivity of TAB ranges from 24% to 77%. However, the results often have a limited impact on clinical management, as a negative result does not definitively rule out the diagnosis [7-10].

The expected positivity rates of temporal artery biopsies and criteria for patient selection are debated in the literature, with no consensus on the definitive diagnostic features. False negatives can occur due to factors such as the timing of the biopsy, the length of the specimen obtained, or the presence of "skip lesions" [3,8,10].

The American College of Rheumatology guidelines for managing giant cell arteritis (GCA) suggest that a temporal artery biopsy (TAB) should be conducted within 14 days of starting glucocorticoid treatment. According to the British Society for Rheumatology (BSR) guidelines, a TAB should be performed by a surgeon skilled in the procedure, and the biopsy samples should be at least 1 cm long after fixation. Additionally, the pathologist assessing the biopsy should be experienced in diagnosing GCA [4,11].

The length of corticosteroid treatment can influence the likelihood of a positive TAB in patients with suspected GCA; however, the biopsy can still be positive for several weeks after starting glucocorticoid therapy. Since the arterial specimen shrinks after excision, a sample size of >10 mm is generally advised to improve the diagnostic yield of TAB. A biopsy on the contralateral side may slightly increase the yield but is usually unnecessary [4,6,12-14].

PMR and GCA are related inflammatory diseases commonly occurring with ageing. Hypertension is considered a risk factor for these conditions, while type 2 diabetes has been reported to offer some protection [15]. We aim to assess the outcomes of our procedural performance according to the British Society for Rheumatology (BSR) guidelines. Additionally, we also evaluated the occurrence of GCA diagnosis with other comorbidities. This article was previously presented as an oral presentation at The West Midlands Surgical Society Conference on May 26, 2023.

## Materials and methods

Study design and setting

A retrospective analysis of prospectively collected data on all patients who underwent temporal artery biopsy while managing giant cell arteritis was conducted from 2017 to 2022 at the University Hospitals of North Midlands, England. The TAB service in the hospital was changed from Ophthalmology to Vascular during pre-COVID.

Data collection

After the audit registration (registration number: CA05623) in the Quality, Safety, and Compliance Department, data on demographics, comorbidities, and pathology reports were extracted from the online database and notes on a pre-defined Excel (Microsoft Corporation, Washington, United States of America) sheet. Clinical letters were also reviewed to gather information.

Outcomes

The primary outcome focused on adherence to BSR guidelines and our local hospital service provisions. These include the time interval from referral to biopsy procedure and the specimen size. Secondary outcomes included examining the relationship between biopsy-confirmed GCA and other comorbidities.

Data analysis

Statistical analysis was carried out using SPSS version 29 (IBM Corporation, Armonk, New York, United States of America). Continuous variables were assessed using two-sample t-test (two-sided) with a 95% confidence interval. Categorical variables were evaluated using the Chi-square/Fisher exact test. The latter was used if more than 20% of cells had an expected count of less than 5. Holm-Bonferroni method was incorporated to adjust for multiple comparisons, and the significance level alpha was set at 5%. The study is reported in line with SQUIRE 2.0 reporting guidelines [16].

## Results

The study included 156 patients with high suspicion of GCA who underwent temporal artery biopsy (TAB). The male-to-female ratio was 0.44:1. The median age was 73. Among the patients, 30 (19%) were smokers. Of the patients, 80 (51%) had right-sided symptoms. Headache was reported in 136 (87%) cases, while vision problems were reported in 12 (7.7%). Mixed symptoms were present in 14 (9%) cases, and atypical presentation was reported in four (2.6%).

Nineteen (12%) patients were on systemic long-term immunosuppressants before being suspected of GCA. Of these, nine (47%) had a positive biopsy result, and the p-value was below the statistically significant threshold (Table 1).

**Table 1:** Giant Cell Arteritis and Disease Association TAB: temporal artery biopsy; PMR: polymyalgia rheumatica; DM: diabetes mellitus.

	TAB positive (45)	TAB negative (111)	p-value	Test statistics
PMR	0.200	0.126	0.318	Fisher's Exact Test
DM	0.089	0.117	0.779	Fisher's Exact Test
Hyperlipidaemia	0.200	0.171	0.671	Pearson's Chi-Square = 0.181
Inflammatory disease	0.133	0.153	0.752	Pearson's Chi-Square = 0.100
Hypertension	0.356	0.351	0.96	Pearson's Chi-Square = 0.002
Hypothyroidism	0.071	0.081	1	Fisher's Exact Test
Immunosuppression	0.200	0.090	0.057	Pearson's Chi-Square = 3.616
Smoking	0.133	0.216	0.234	Pearson's Chi-Square = 1.416

**Figure 1:**
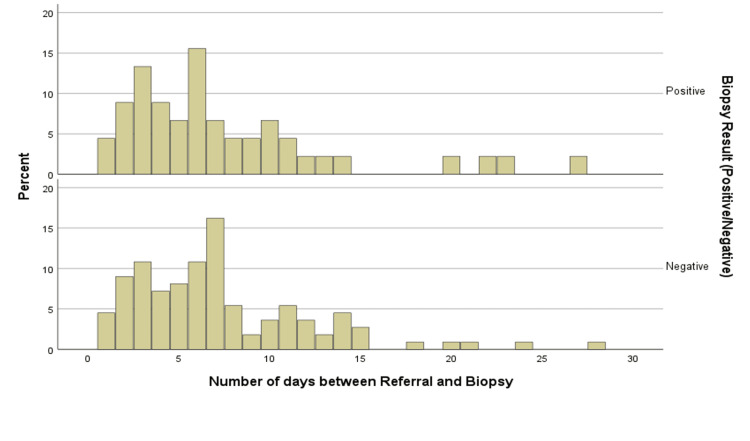
Time of Referral to the Biopsy Procedure

The mean interval between the time of referral to the biopsy procedure was 7.4 days (Figure 1).

A total of 103 (66%) TAB were carried out within seven days of starting steroids; of these, 29 (28%) were positive for GCA. On the other hand, analysis showed that 41 (26%) biopsies were performed within 14 days of referral. Of these, 12 (29%) specimens were positive for GCA. Due to several reasons, 12 (8%) performed beyond 14 days with positive in four (33%). Overall, positive biopsy results were found in 45 (29%) cases.

The procedures were carried out by either a vascular surgeon 119 (76%) or an ophthalmologist 37 (24%). The consultants performed most of the procedures in 143 (92%) cases. In 73 (47%) cases, the post-fixation biopsy sample size was greater than 10 mm. Overall, positive biopsy results for GCA were found in 45 (29%) patients, and the biopsy outcome was not dependent on the speciality, time from referral to biopsy, nor on the length of the post-fixation specimen (Table 2).

**Table 2:** Biopsy Outcome vs Time From Referral to Biopsy, Length of the Post-fixation Specimen, and the Speciality TAB: temporal artery biopsy.

	TAB positive (45)	TAB negative (111)	p-value	Test statistics
Interval between referral and biopsy (days)	7.53±5.915	7.28±4.958	0.785	t-value = -0.274
Specimen length <7	0.222	0.216	0.926	Pearson's Chi-Square = 0.154
Specimen length 7-9	0.333	0.306		
Specimen length >10	0.444	0.477		
Specialty of the surgeon (Vascular vs Ophthalmology)	0.800	0.748	0.487	Pearson's Chi-Square = 0.483

GCA was confirmed in nine (39%) patients with polymyalgia rheumatica, four (24%) with diabetics, three (20%) with hypothyroidism, 16 (29%) with hypertension, nine (32%) with hyperlipidaemia, and six (26%) with other inflammatory diseases. The biopsy results did not vary significantly across these conditions (Table 1).

## Discussion

Giant cell arteritis (GCA) is most prevalent in northern European countries and more common in southern areas of the UK [1]. Our hospital is a tertiary care teaching hospital in the North Midlands of England and serves a population of approximately three million people. Initially, the temporal artery biopsy (TAB) service was managed by the Ophthalmology department. However, pre-COVID-19, this responsibility shifted to the vascular department, resulting in a higher number of TABs performed by vascular surgeons compared to ophthalmologists. In 73 cases (47%), the post-fixation biopsy sample size exceeded 10 mm.

The British Society for Rheumatology (BSR) updated its guidance and recommended that TABs be conducted by a surgical unit experienced in the procedure, with specimens at least 10 mm long. A positive temporal artery biopsy demonstrating inflammatory features characteristic of GCA, such as giant cells or panarteritis, definitively confirms the diagnosis of giant cell arteritis. However, the true sensitivity of temporal artery biopsy is less than 100%, a limitation underscored by the histological presence of skip lesions observed in some cases, leading to false-negative histopathology. A study by Muratore et al., which reported a 35% GCA positivity rate, suggested that a post-fixation biopsy specimen length of 5 mm is sufficient for diagnosis if the patient is accurately selected [17]. Similarly, research by Butendieck et al. found no significant impact of specimen length on the positivity rate [4,10,17,18]. Our study also found no significant difference between the length of the post-fixation specimen and positive results.

According to the British Society for Rheumatology (BSR) guidelines, any patient over 50 with a sudden onset of headache, visual disturbances, and jaw claudication should be suspected of having giant cell arteritis (GCA). The incidence of visual disturbances in patients with GCA has reportedly declined [4,19]. In our cohort, the headache was the most common symptom, followed by a visual disturbance in less than 8% of cases. GCA seldom occurs before the age of 50, with the incidence rising steadily after that and peaking between the ages of 70 and 79 [19]. In our study, the most common age group was also between 70 and 79 years.

Temporal artery biopsy (TAB) is a minimally invasive procedure performed under local anaesthesia with a low risk of complications. TAB remains positive for up to six weeks, although sensitivity may decrease over time. This is why TAB is recommended for diagnosing GCA. The specificity of TAB is up to 100%, while pooled sensitivity is reported at 77%. The literature indicates that inflammation associated with GCA can persist despite steroid therapy. It is recommended that referring physicians include the dosage and initiation date of steroid therapy as essential information on the request form [4,8-9,19]. In our cohort, most TABs were performed within seven days, with a mean interval from referral to biopsy of 7.4 days. However, the histopathology results were not dependent on length and timing.

In the Western elderly population, the presentation of GCA commonly overlaps with polymyalgia rheumatica (PMR), with hypertension identified as a risk factor. The incidence of diabetes is higher, possibly due to long-term steroid use. Other related comorbidities include hyperlipidaemia, thyroid disorders, cerebrovascular diseases, and osteoporosis. A study by Brennan et al. confirmed a statistically significant increased risk of GCA in current or former smokers [19-23]. In our study, 39% of patients had a history of PMR, 32% had hyperlipidaemia, 29% had hypertension, 20% had hypothyroidism, 19% were smokers, and 12% were on long-term systemic immunosuppressants. However, histopathology results did not vary significantly across these patient groups.

Literature indicates that in patients with suspected GCA, the positive rate of biopsy specimens is 25%-35%. A recent study from South Yorkshire, UK, by Ling and Carter showed a positivity rate of 31.3% [1,21]. Our overall positive biopsy rate for GCA was 29%, consistent with the existing literature.

This study is based on retrospective analysis of prospectively collected data in keeping with National Rheumatology Guidelines (BSR guidelines) [4] for the surgeons to change the practice. However, we do recognise that our study is a small, single-centre retrospective analysis on TAB performed on suspected cases of GCA referred by British rheumatologists, which may not represent the entire population. The retrospective nature of this study is also constrained by the data collected from the online documentation system, which means we relied on the clerking and coding of the admitting team rather than actively querying patients about comorbidities, and this might have impacted on the results. Conducted in one tertiary hospital in England, this study may be subject to selection bias, potentially limiting its statistical strength. Additionally, we did not consider the grade of the pathologist who reported the results, which could have impacted the findings.

## Conclusions

Temporal artery biopsy remains a valuable and crucial diagnostic tool in challenging equivocal cases of giant cell arteritis (GCA), although it is limited by its sensitivity. There is still uncertainty regarding the relationship between biopsy positivity, post-fixation sample size, and the interval between referral and procedure. Additionally, the speciality of the clinician performing the biopsy does not appear to significantly influence the likelihood of a positive result. In the limited sample, finding the relationship between concomitant comorbidities and GCA is not feasible, although literature supports it. Despite this, there is always room to improve. Given the current positivity rates and disease associations, we suggest a larger multicentre randomised trial to unanswered questions.

## References

[1] Ling WN, Carter S (2023). Demographic characteristics of patients with giant cell arteritis in Sheffield, England. Clin Med (Lond).

[2] Saedon H, Saedon M, Goodyear S, Papettas T, Marshall C (2012). Temporal artery biopsy for giant cell arteritis: retrospective audit. JRSM Short Rep.

[3] Villeneuve E, Lacroix JM, Brisebois S (2023). Optimizing the use of temporal artery biopsy: a retrospective study. J Otolaryngol Head Neck Surg.

[4] Mackie SL, Dejaco C, Appenzeller S (2020). British Society for Rheumatology guideline on diagnosis and treatment of giant cell arteritis. Rheumatology (Oxford).

[5] Mackie SL, Dasgupta B, Hordon L (2011). Ischaemic manifestations in giant cell arteritis are associated with area level socio-economic deprivation, but not cardiovascular risk factors. Rheumatology (Oxford).

[6] Chatzigeorgiou C, Taylor JC, Elliott F, O'Sullivan EP, Morgan AW, Barrett JH, Mackie SL (2023). Common co-morbidities in polymyalgia rheumatica and giant cell arteritis: cross-sectional study in UK Biobank. Rheumatol Adv Pract.

[7] Davies C, Frost B, Eshan O, McLain AD, Shandall A (2006). Temporal artery biopsy...who needs one?. Postgrad Med J.

[8] Taze D, Chakrabarty A, Venkateswaran R (2024). Histopathology reporting of temporal artery biopsy specimens for giant cell arteritis: results of a modified Delphi study. J Clin Pathol.

[9] Rubenstein E, Maldini C, Gonzalez-Chiappe S, Chevret S, Mahr A (2020). Sensitivity of temporal artery biopsy in the diagnosis of giant cell arteritis: a systematic literature review and meta-analysis. Rheumatology (Oxford).

[10] Jiang Z, Ji H, Dong J (2023). Temporal artery biopsy for suspected giant cell arteritis: a mini review. Indian J Ophthalmol.

[11] Maz M, Chung SA, Abril A (2021). 2021 American College of Rheumatology/Vasculitis Foundation Guideline for the management of giant cell arteritis and Takayasu arteritis. Arthritis Rheumatol.

[12] Papadakos SP, Papazoglou AS, Moysidis DV (2023). The effect of corticosteroids on temporal artery biopsy positivity in giant cell arteritis: timing is everything. J Clin Rheumatol.

[13] Mohammad AJ, Englund M, Turesson C, Tomasson G, Merkel PA (2017). Rate of comorbidities in giant cell arteritis: a population-based study. J Rheumatol.

[14] Jakobsson K, Jacobsson L, Mohammad AJ, Nilsson JÅ, Warrington K, Matteson EL, Turesson C (2016). The effect of clinical features and glucocorticoids on biopsy findings in giant cell arteritis. BMC Musculoskelet Disord.

[15] Dasgupta B, Borg FA, Hassan N (2010). BSR and BHPR guidelines for the management of giant cell arteritis. Rheumatology (Oxford).

[16] Ogrinc G, Davies L, Goodman D, Batalden P, Davidoff F, Stevens D (2016). SQUIRE 2.0 (Standards for QUality Improvement Reporting Excellence): revised publication guidelines from a detailed consensus process. BMJ Qual Saf.

[17] Muratore F, Boiardi L, Cavazza A (2021). Association between specimen length and number of sections and diagnostic yield of temporal artery biopsy for giant cell arteritis. Arthritis Care Res (Hoboken).

[18] Butendieck R Jr, Calamia K, Sandin A (2023). A study of temporal artery biopsy for the diagnosis of giant cell arteritis. Clin Rheumatol.

[19] Singh AG, Kermani TA, Crowson CS, Weyand CM, Matteson EL, Warrington KJ (2015). Visual manifestations in giant cell arteritis: trend over 5 decades in a population-based cohort. J Rheumatol.

[20] Gonzalez-Gay MA, Vazquez-Rodriguez TR, Lopez-Diaz MJ, Miranda-Filloy JA, Gonzalez-Juanatey C, Martin J, Llorca J (2009). Epidemiology of giant cell arteritis and polymyalgia rheumatica. Arthritis Rheum.

[21] Monti S, Schäfer VS, Muratore F, Salvarani C, Montecucco C, Luqmani R (2023). Updates on the diagnosis and monitoring of giant cell arteritis. Front Med (Lausanne).

[22] Tomelleri A, van der Geest KS, Khurshid MA (2023). Disease stratification in GCA and PMR: state of the art and future perspectives. Nat Rev Rheumatol.

[23] Brennan DN, Ungprasert P, Warrington KJ, Koster MJ (2018). Smoking as a risk factor for giant cell arteritis: a systematic review and meta-analysis. Semin Arthritis Rheum.

